# Important factors affecting red blood cell distribution shouldn't be ignored

**DOI:** 10.1080/0886022X.2022.2110895

**Published:** 2022-08-15

**Authors:** Yu Zhao, Wenyun Wang, Zhilong Dong

**Affiliations:** a Department of Medicine, Northwest University for Nationalities, Lanzhou, PR China; b Department of Pediatric Surgery, Second Hospital of Lanzhou University, Lanzhou, PR China; c Department of Urology, Second Hospital of Lanzhou University, Lanzhou, PR China

Dear Editor,

The paper, which entitled ‘Red blood cell distribution width at admission outcome in critically ill patients with kidney failure: a retrospective cohort study based on the MIMIC-IV database’ by Hua et al. [1], is of great interest to us. The retrospective study included 674 kidney failure patients (643 hemodialysis and 31 peritoneal dialysis patients), who were divided into three groups based on tertiles of red blood cell distribution width (RDW) {the low group: <15.4% (*n* = 223); the middle group: ≥15.5%, <17.1% (221); the high group: ≥1.72%(*n* = 230)}. The Kaplan-Meier analysis showed that elevated RDW (≥15.5%) had a lower survival rate. The Cox regression model indicated that middle and high levels of RDW were associated with an increased risk of ICU all-cause mortality (HR: 3.81 and 4.71, *p* < 0.05, respectively), 30-day all-cause mortality (HR: 2.85 and 6.62, *p* < 0.05, respectively), 180-day all-cause mortality (HR: 2.55 and 4.43, *p* < 0.05, respectively), and 1-year all-cause mortality (HR: 2.51 and 4.08, *p* < 0.05, respectively) in critically ill patients with kidney failure after adjusting for potential confounders in model II. Subgroup analysis showed that RDW was a risk factor in all of there interactive stratifications in Table 3. The paper found that the value of RDW could provide useful information for risk classification to clinicians, and elevated levels of RDW were associated with an increased risk of all-cause mortality in critically ill patients with kidney failure. I pay special attention to the laboratory results of this research because it caused me some concerns.

RDW has emerged as a novel prognostic marker for serious adverse events in recent decades. Malnutrition is common in patients on dialysis in terms of dialysis-induced nutrient losses and a low protein diet [2] and has been proposed as possible etiology about the underly mechanism between higher RDW levels and elevated all-cause mortality of patients with kidney failure. Firstly, a restrospective observational study conducted by Tania et al. [3]. 109,675 adult maintenance HD patients with baseline and time-varying RDW showed that higher RDW (≥15.5%) is associated with incrementally higher mortality risk. Correlation and liner regression analysis showed that every 1 g/dL increase in albumin level, RDW decreased by 0.731% (correlation coefficient, −0.20).

Secondly, Soohoo et al. [4]. conducted a large cohort study of 14,323 PD patients with a mean RDW of 15.3 ± 1.6% to show the higher baseline and time-varying RDW (≥15.0%) were associated with a greater risk of mortality, including all-cause and CV, and time to first hospitalization. Subgroup analysis showed that higher RDW (≥15.0%) was not significantly associated with all-cause mortality among patients with iron saturation (ISAT) ≥30%. However, neither nutritional data (e.g., serum levels of folate, vitamin B12, and serial iron profifiles) nor of bone marrow function parameters (e.g., reticulocyte and platelet count) nor blood transfusion data and erythropoietin-stimulating agent (ESA) use and dosage were routinely examined in the paper by Hua et al. [1].

Thirdly, a large population-based retrospective cohort study of 3,156,863 adults from the general population by Tonelli et al. [5]. evaluated the association of RDW and standard deviation of red blood cell size (SD-RBC: calculated from the product of RDW and mean corpuscular volume) with the risk of adverse outcome for a median follow-up of 6.8 year. The paper used Cox regression to determine the association between baseline RDW and SD-RBC percentiles (<1, 1–5, 5–25, 25–75, 75–95, 95–99, >99) and the first occurrence of each clinical outcomes during follow-up. The association between SD-RBC and mortality was similar to that between RDW and mortality. The values of RDW for the risk of ESRD (initiation of renal replacement therapy) were not associated in the <25th percentiles but higher for participants in the 75th–95th percentiles (HR 1.15, *p* < 0.05), the 95th–99th percentiles (HR 1.29, *p* < 0.05) and the 99th percentile (HR 1.05, *p* > 0.05), the association between RDW and ESRD was progressively attenuated with further adjustment for confounders and was not observed in the fully adjusted model. The lower (<25th percentile) values of SD-RBC were strongly associated with ESRD in Model 3. The higher values of SD-RBC for participants in the 95th–99th percentiles (HR 1.35, *p* < 0.05) and the 99th percentile (HR 1.38, *p* < 0.05) were associated with excess risk of ESRD in the fully adjusted model. Finding that SD-RBC is stronger than RDW for all associations, and both lower (<25th percentile) and higher (>95th percentile) values of SD-RBC were independently associated with ESRD. I think SD-RBC may be useful as prognostic marker for serious adverse events.

Finally, I found some problems with the sequence of references from 14 to 17 that were inconsistent with the content of the paper [1]. Therefore, the accuracy of the reference has yet to be further verified by the author.
